# Detection and mapping of homologous and homoeologous segments in homoeologous groups of allotetraploid cotton by BAC-FISH

**DOI:** 10.1186/1471-2164-8-178

**Published:** 2007-06-19

**Authors:** Kai Wang, Wangzhen Guo, Tianzhen Zhang

**Affiliations:** 1 National Key Laboratory of Crop Genetics and Germplasm Enhancement, Cotton Research Institute, Nanjing Agricultural University, Nanjing 210095, China

## Abstract

**Background:**

Cotton, as an allopolyploid species, contains homoeologous A and D subgenomes. The study of the homoeologous (duplicated) segments or chromosomes can facilitate insight into the evolutionary process of polyploidy and the development of genomic resources. Fluorescence *in situ *hybridization (FISH) using bacterial artificial chromosome (BAC) clones as probes has commonly been used to provide a reliable cytological technique for chromosome identification. In polyploids, it also presents a useful approach for identification and localization of duplicated segments. Here, two types of BACs that contained the duplicated segments were isolated and analyzed in tetraploid cotton by FISH.

**Results:**

Homologous and homoeologous BACs were isolated by way of SSR marker-based selection and then used to develop BAC-FISH probes. Duplicated segments in homoeologous chromosomes were detected by FISH. The FISH and related linkage map results followed known reinforced the relationships of homoeologous chromosomes in allotetraploid cotton, and presented a useful approach for isolation of homoeologous loci or segments and for mapping of monomorphic loci. It is very important to find that the large duplicated segments (homologous BACs) do exist between homoeologous chromosomes, so the shot-gun approach for genome sequencing was unavailable for tetraploid cotton. However, without doubt, it will contain more information and promote the research for duplicated segments as well as the genome evolution in cotton.

**Conclusion:**

These findings and the analysis method by BAC-FISH demonstrated the powerful nature and wide use for the genome and genome evolutionary researches in cotton and other polyploidy species.

## Background

Polyploidy is an evolutionary process whereby two or more genomes are brought together in the same nucleus, usually by hybridization followed by chromosome doubling [1]. Accordingly, most of these genomes contain duplicated chromosomes or chromosomal segments that reflect ancient or recent rounds of polyploidy. Therefore, investigations of the levels of diversity and patterns of duplicated genes and segments in polyploid plants can provide insights into the process of polyploidization and subsequent processes. When genes are duplicated as a consequence of polyploidization, they may continue to evolve at the same rate as they did in their diploid ancestors, or they may be subject to pressures that lead to differential rates of sequence evolution [2]. Ultimately, these duplicated sequences and their functions are maintained intact or undergo long-term evolutionary change via sequence elimination [3,4], sequence rearrangement [5], gene silencing [6], or acquisition of new function [7]. Most of the evolution process of large segments or genome is accompanied with the duplicated genes evolution or operates organizational level of duplicated genes. The process of polyploidy evolution leading to stabilization and species formation have been studied and confirmed by modern molecular genetic techniques [3,8-11]. A new phenomenon occurring after polyploidy was found by analysing the evolution of dispersed repeats, and the results showed that there has been substantial colonization of the D genome by A genome repetitive elements [10,12]. The various aspects of genome evolution involving duplicated sequences in polyploids have been reviewed elsewhere [2].

Cotton (*Gossypium*) is particularly useful for studies of polyploidy [13]. A simple method for isolating homoeologous loci from allopolyploids has been developed in cotton [14]. In addition, investigations of duplicated genes have revealed their evolutionary rate [15], patterns and levels of nucleotide diversity [16-18], and functional silencing [19] exerted polyploidy. Nevertheless, the relatively few examples studied to date provide little understanding of genomic evolution after duplication. In fact, reports on evolutionary rate have been contradictory [15,17], probably due to limited sampling.

In this study, different types of duplicated segments-containing bacterial artificial chromosomes (BACs) were isolated. Fluorescence *in situ *hybridization (FISH), performed with a complete set of chromosome-specific BAC clones developed in tetraploid cotton [20], discriminated the duplicated segments locating on homoeologous chromosomes. The results present new evidence for tetraploid cotton homoeologous chromosomes relationship as well as a new approach to the isolation of homoeologous loci or segments. And these also provided a new chance for the research of duplicated sequences in cotton.

## Results

### Homoeologous BACs derived from homoeologous chromosomes

**Figure 1:**
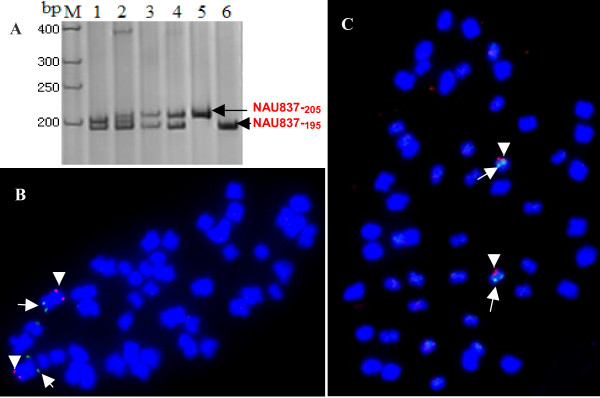
**Distribution analysis of homoeologous segments in tetraploid cotton by FISH**. (A) Figure showed that the identification of the BAC clone 75F07 (lane 5) containing the polymorphic locus of NAU837_-205_, and 68D15 (lane 6) containing the monomorphic locus NAU837_-195 _between TM-1 and Hai7124 by SSR marker NAU837. Lanes 1–4 were Hai7124, F_1_(TM-1 × Hai7124), TM-1 and restorer line 0-613-2R, respectively. (B) FISH image showed that the signals of the polymorphic allele BAC 75F07 (green signals, arrows) and chromosome A6-specific BAC 47N15 (red signals, arrowheads) were located on the same chromosome. It indicated that the polymorphic allele BAC 75F07 derived from chromosome A6. (C) FISH image showed that the BAC clone 68D15 (red signals, arrowheads) located on the same chromosome with chromosome-specific BAC clone 24K19 (green signals, arrows) of chromosome D6.

SSR primer pair NAU837 yielded two PCR amplicons from *G. barbadense *cv. Hai7124 and two from *G. hirsutum *acc. TM-1 (Figure 1A). One produced polymorphic alleles, NAU837_-205 _in Hai7124 and NAU837_-215_in TM-1, and was further mapped on chromosome A6. However, both Hai7124 and TM-1 produced a same large fragment, monomorphic locus NAU837_-195 _which could not be mapped to its corresponding chromosome using the present molecular tagging strategy. This is very common in tetraploid species. Where is such monomorphic locus located in cotton? Since cotton is an allotetraploid, it is supposed that the locus is located in the homoeologous chromosome of A6, i.e. chromosome D6. Two positive BAC clones, 75F07 and 68D15, were identified by screening the BAC libraries with NAU837. The 75F07 clone was the positive clone of polymorphic locus NAU837_-205_, and 68D15 was the positive clone of the monomorphic locus NAU837_-195_. These two BACs were FISHed simultaneously to determine if they were located on the same chromosome. The result showed that they were located on two different pairs of chromosomes (data not shown). As expected, BAC 75F07 mapped to a pair of larger chromosomes, while BAC 68D15 mapped to another pair of smaller chromosomes that we speculated belonging to the D subgenome. FISH with the chromosome A6-specific BAC 47N15 [21] confirmed that the polymorphic locus derived-BAC 75F07 was still physically located on chromosome A6 (Figure 1B, arrowhead). Additionally, simultaneous FISH of the monomorphic locus derived-BAC 68D15 with chromosome D6-specific BAC 24K19 showed that they are both located on chromosome D6 (Figure 1C). This result indicated that the monomorphic locus was most likely derived from the homoeologous chromosomes.

**Figure 2:**
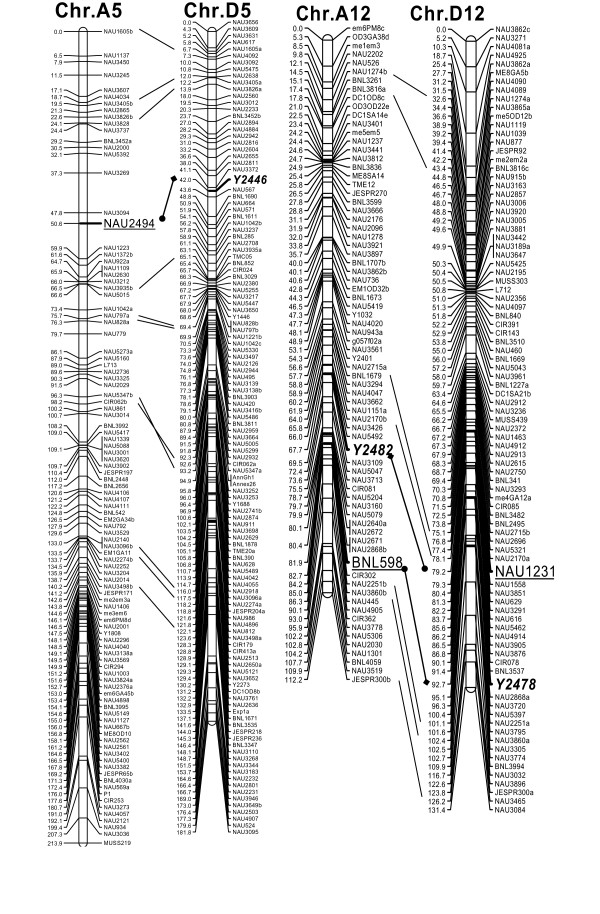
**The linkage mapping of monomorphic locus-derived markers**. The monomorphic BAC-derived markers Y2446, Y2482 and Y2478 were indicated in bold italics, and linked with the corresponding polymorphic loci which were underlined. Other duplicated loci were also connected by solid bar between the homoeologous chromosomes.

More BACs were chosen to determine if derivation from homoeologous chromosomes is a common phenomenon. Five other BACs 66B19, 70L23, 78J02, 38P15, and 84A20 were isolated as monomorphic loci between TM-1 and Hai7124. These five BACs were identified using SSR markers NAU921, BNL598, NAU877, NAU1231 and NAU2494, respectively. Their corresponding polymorphic loci, NAU921_-400_, BNL598_-123_, NAU877_-205_, NAU1231_-250_, and NAU2494_-215 _had been mapped to chromosomes A10, A12, D12, D12, and A05. To test whether they were truly derived from corresponding homoeologous chromosomes (i.e., chromosomes D10, D12, A12, A12, and D05), BACs 66B19, 70L23, 78J02, 38P15, and 84A20 were directly FISHed with their corresponding homoeologous chromosome-specific BACs, 78O17, 10G17, 43C02, 43C02, and 50D03 [21]. The results showed that except BAC 84A20 which could not generate clear signals, all of the monomorphic locus BACs (66B19, 70L23, 78J02, and 38P15) were mapped to the corresponding homoeologous chromosomes D10, D12, A12, and A12 (Table 1) (Figure not shown). Furthermore, both ends of all these seven BACs including 75F07 and 68D15 were sequenced, and specific primers were designed. Among them, three pairs of primers, Y2478, Y2482 and Y2446 (Table 2), were polymorphism between TM-1 and Hai7124 which were two parents of BC_1 _mapping population [22]. And these three loci derived from the monomorphic loci BACs were mapped exactly on the corresponding homoeologous chromosomes (Table 1, Figure 2). The homology detected for one pair of BACs that originated from homoeologous chromosomes reinforced previous results with homoeologous chromosomes, and was novel evidence for relationship identification of homoeologous chromosomes in allotetraploid cotton.

**Table 1:** Distribution analysis of homoeologous SSR markers

**Polymorphic loci**	**Genetic mapping of polymorphic loci**	**Monomorphic loci**	**Monomorphic loci BACs**	**BAC end primers**	**Physical location of monomorphic loci BACs**	**Linkage mapping of BACs**
NAU921_-400_	A10	NAU921_-200_	66B19	-	D10	-
NAU877_-205_	D12	NAU877_-215_	78J02	-	A12	-
BNL598_-123_	A12	BNL598_-115_	70L23	Y2478	D12	D12
NAU1231_-250_	D12	NAU1231_-230_	38P15	Y2482	A12	A12
NAU2494_-215_	A05	NAU2494_-180_	84A20	Y2446	-	D05

**Table 2:** Polymorphic primers and corresponding BAC end sequences used in this study

**BAC clones**	**GenBank accession number of end sequences**	**Primers**	**Primer sequences**
70L23	EF182757	Y2478	5'-ATCGGAGCTCCATTAACAAA-3'
			5'-GCCACCACGTCTCTATTTTT-3'
38P15	EF182758	Y2482	5'-TGTTGGACCTTTCTCCAAAT-3'
			5'-GCTAAAGCAGACGTATACGAAA-3'
84A20	EF182756	Y2446	5'-TGGCAGAAACCTAATTCTAGC-3'
			5'-AAAAATCTGCAGTTGCCTTC-3'

### Homologous BACs derived from homoeologous chromosomes

**Figure 3:**
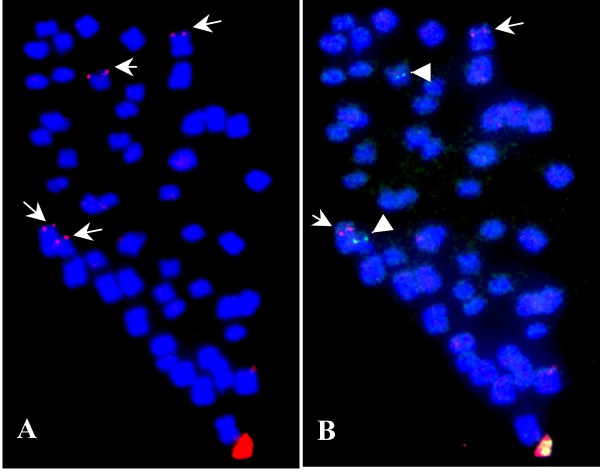
**Distribution analysis of homologous segments in tetraploid cotton by FISH**. (A) BAC clone 09D09 produced two pairs of bright signals (arrows) in tetraploid cotton mitotic cell. (B) repeated-FISH showed the locations of two chromosome-specific probes 87P01 (red signal, arrows) of chromosome A5 and 50D03 (green signal, arrowheads) of chromosome D5. The results showed that the signals produced by clone 09D09 were located on the homoeologous chromosomes A5 and D5.

The use of BAC clone 09D09, isolated from the TM-1 library, in FISH analysis led to the interesting finding that large homologous segments do exist between homoeologous chromosome pairs. This BAC clone was isolated with SSR marker BNL3452_-180 _during the isolation of chromosome-specific BAC clones [20]. Because BAC 09D09 generated two different signals on two pairs of chromosomes, it could not be used as a chromosome-specific BAC. As shown in Figure 3A, bright signals were clearly detected on two pairs of chromosomes. A reasonable explanation for the double signal is that large duplicated segments in allotetraploid cotton are maintained during long-term evolution after polyploidy formation. If this is the case, they should be located on one pair of homoeologous chromosomes [15,17,18]. Therefore, repeated-FISH was carried out to test the locations of these two signals. Because the BNL3452_-180 _locus has been genetically mapped to chromosome D5 [22], the chromosome-specific BACs 87P01 (A5) and 50D03 (D5) [21] were simultaneously re-hybridized on the same slide as Figure 3A. A comparison between Figure 3A and 3B clearly showed that the two pairs of signals produced by BAC 09D09 (Figure 3A) were located on the A5/D5 pair of homoeologous chromosomes (Figure 3B). The equal strength of the FISH signals confirmed our supposition that BAC 09D09 contains a copy of a duplicated homologous DNA segment from homoeologous chromosomes A5 and D5, and that homologous segments are present in the allotetraploid.

BAC-FISH of another BAC clone, 68O15, further supported these findings. This BAC was isolated using NAU2195_-200_, which mapped on chromosome D12. Like BAC 09D09, it also generated two pairs of bright signals on two pairs of different chromosomes. Repeated-FISH with A12 and D12 chromosome-specific BACs, 43C02 and 10G17, found that these two pairs of signals originated from the corresponding homoeologous chromosomes A12 and D12 (figures not shown).

**Figure 4:**
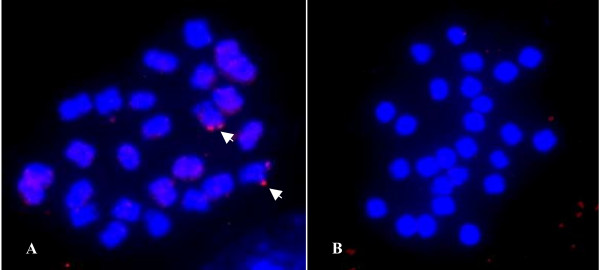
**FISH photomicrographs of *G. hirsutum *duplicated segments hybridized to metaphase chromosomes of *G. arboreum *and *G. raimondii***. BAC clone 09D09 produced one pair of signals (arrows) on one pair of mitotic chromosomes of *G. arboreum *(A), but no signals on the chromosomes of *G. raimondii *(B).

To further evaluate the physical distribution of duplicated segments in diploid cotton, the *G. arboreum *(A_2_) and *G. raimondii *(D_5_) chromosomes were used in FISH with BAC clone 09D09. As shown in Figure 4A, only the A-genome *G. arboreum *contained the duplicated segments, with no signal from the D-genome *G. raimondii *(Figure 4B). It indicated that this duplicated segments within BAC clone 09D09 originated from the A genome and has "jump" to the D subgenome after the polyploidy formation in cotton (detailed in Discussion).

## Discussion

### Identification and analysis of homoeologous chromosome BACs

Tetraploid cotton (n = 2x = 26, AD) was derived from two diploids with A and D genomes that diverged from a common ancestor [23]. Therefore, in theory, there should be 13 pairs of homoeologous chromosomes in tetraploid cotton. Recently, by distribution analysis of duplicated marker loci among chromosomes, all 13 pairs of homoeologous chromosomes have been discriminated [24,25]. Here, these findings of duplicated segments derived from BACs provide new evidence for homoeologous chromosome discrimination. As shown in Figure 1A and 1B, two BACs derived from the monomorphic and polymorphic loci of one marker were identified on the A6/D6 homoeologous chromosomes which had been identified based on the similar phenotypes of monosomic plants [26] and duplicated molecular loci. For each BAC, the same primer origin indicated the existence of homologous sequences, and the distribution of duplicated segments confirmed the homoeologous relationships. So it was considered as well homoeologous segments (BACs) as new evidences for homoeologous chromosome detection.

This finding could be applied to the high-density physical map construction of the cotton genome. As demonstrated above, the two bands (two loci) produced by marker NAU837 which likely derived from the homology between different homoeologous chromosomes in allotetraploid cotton. Therefore, one of this kind of markers could be used to screen two groups of BACs that are located on one pair of homoeologous chromosomes. In fact, it effectively increased by one fold the number of BACs for physical map construction. It is particularly useful since this kind of BAC can not be isolated using the molecular loci on a genetic map due to the monomorphism between mapping parents. Therefore, these BACs are needed for the continued construction of the physical map and for further physical map based-genome sequencing. It has been used to help isolate cotton BACs for contigs construction in our lab. Furthermore, the genetic location of BAC-ends sequences presents a test for the location of monomorphic locus BACs. On the other hand, it also demonstrated an alternative mapping and researching approach for this kind of monomorphic locus which usually occurred in genetic marker and could not mapped by the means of genetic mapping. It makes the further researches of this kind of duplicated loci possible, and the collinearity analysis of duplicated loci produced by some EST-derived SSR also has been carried out in our lab to discover the evolution of duplicated genes in tetraploid cotton.

Cotton is a naturally occurring polyploid with eight diploid genomes distributed over four continents [2]. And it has become a useful model system for the study of the genome and molecular evolution of allopolyploids, especially with the modern molecular evolution researches on duplicated loci [15,16,27-29]. Here, our finding provided a novel approach for duplicated loci isolation that avoids the likelihood of errors from PCR amplification [30]. More importantly, due to the normal phenomenon of markers like NAU837 in tetraploid cotton, the large scale isolation of duplicated loci is much simpler. The isolation and testing of a sufficient number of duplicated loci will unravel some previous contradictories [15,17,18] and provide the basis for phylogenetic analysis of lineages. And the finding of duplicated segments in BACs would also provide new challenges in the study of cotton genome evolution by analyzing its distribution, maintenance mechanisms during long periods of diploidization, and diversity following polyploidization.

### Homologous BAC identification and analysis

Another case showed as BAC 09D09 is similar to the duplicated molecular loci derived from one molecular marker, and duplicated segments (loci) also discriminated the homoeologous relationship in tetraploid cotton. However, unlike the duplicated molecular loci, most of which are no more than 1 kb, BAC-FISH reveals a much larger segment (~100 kb) of homeology. Therefore, they are more reasonable for finding homoeologous relationships [31]. Several cases were found in which two pairs of signals were detected, but usually one pair of signals was brighter and the other was too weak to be located by multi-FISH. However, one pair of chromosomes was always clearly larger than the other, indicating that they should belong to different subgenomes. Furthermore, the several kb of DNA sequence in FISH detectable signals suggests that the homologous segments likely originate from the homoeologous chromosomes. Therefore, the possibility is raised that in any particular case, the FISH signal derived from a BAC in a different subgenome may reinforce the homoeologous relationships in tetraploid cotton rather than random repetitive-sequence or reciprocal or non-reciprocal translocations.

In comparison with the homeology between the two BACs derived from marker NAU837, which may be just several hundreds of base pairs due to SSR amplification, the duplicated segments originating from BAC 09D09 may be more than 100 kb. Furthermore, the equal strength of the two pairs of FISH signals is indicative of a high level of homology between the segments in the A and D subgenomes. If this degree of homology is the norm, shotgun sequencing is not feasible for tetraploid cotton because the assemblage of such large scale homology would be impossible. With the rapid development of the cotton genetic map and BAC library construction, genome sequencing based on the physical map may prove to be preferred choice for cotton.

Nevertheless, the case of BAC 09D09 is an excellent example of homoeologous analysis because the length of the duplicated segment must be as long as several scores of kilobase pairs due to the bright FISH signals detected. It will, no doubt, provide more information for evolutionary analysis of duplications at the chromosomal or genomic levels. The distribution of BAC 09D09 in the A-genome and not the D-genome of diploid cotton indicated that it originated in the A-genome ancestor and spread to the D-subgenome after polyploidy. This phenomenon was previously described by Hanson et al. (1998) [12] and Zhao et al. (1998) [10] from the analysis of distributions of dispersed repetitive DNA in cotton. And since polyploidization there has been substantial colonization of the D genome by A genome repetitive elements [2]. Our results reinforce the model that A-genomic DNA "infected" the D-subgenome, with homoeology occurring subsequent to polyploidization. However, these findings also point out that large DNA segments do spread to new subgenomes and are maintained during long period of evolution in allotetraploid cotton. It is a very different pattern in DNA content and component from the repetitive DNA theory, so the most probable mechanism may be inter-genomic recombination and exchange between subgenomes in the nonfunctional segments. Because the segments are so large, it is impossible for them to behave as a functional gene or transposon to "jump" between different subgenomes. The concept that pairing and recombination between homoeologous chromosomes does not appear to have substantially affected the organization of the modern cotton genome, as determined by analysis of molecular markers repetitive DNA [12,31], would be novel and contrary to current belief. This novel concept might also provide new opportunities to understand the tetraploid cotton genome and its evolutionary process.

Finally, BAC-FISH mapping presents a powerful tool for evolution research in tetraploid cotton. Previous studies have shown that a correlation exists between chromosomal position and levels of genetic diversity at a locus [32]. However, researchers can not analyze the diversity level of genes or sequences by combining the genetic and physical data because it is not possible to physically map short duplicated genes or sequences, and only the genetic position allows them to reference and speculate in their analysis [18]. We are currently able to correlate these genetic mapping data with physical chromosomal locations constructed by BAC-FISH. Therefore, analysis of the relationship between genetic diversity and chromosomal position will become available for tetraploid cotton.

## Conclusion

In conclusion, BAC-FISH as a new approach for the duplicated segments analysis and physical mapping of monomorphic locus in cotton were presented. Two types of BACs containing duplicated segments were found and physically mapped. The results showed that they were derived from the homoeologous chromosomes. According to the degree of duplicated segment containing in BACs, they were named homologous and homoeologous BACs. Analysis of these BACs presents us some new approach for the researches of cotton genome and genome evolution. Especially, the large duplicated segments (homologous BACs) were identified by FISH, and it demonstrated the powerful potential for the research of cotton genome evolution. In a word, these findings are just an entrance and a challenge for the cotton genome researches.

## Methods

The BACs used in this study were found in two genomic BAC libraries derived from the tetraploid cotton strain TM-1 and 0-613-2R, a restorer line of cytoplasmic male sterility [33]. The TM-1 library was kindly supplied by Dr. John Yu, USDA-ARS, Crops Germplasm Research Unit, College Station, Texas. TM-1 is a highly inbred line of *G. hirsutum *L. (2n = 52). The A and D subgenome chromosomes were renamed A1 through A13 and D1 though D13, respectively, based on new tetraploid cotton nomenclature for homoeologous chromosomes [20].

Only TM-1 mitotic metaphase chromosomes were used for FISH. Root tip, slide preparation, and single-color FISH have been described previously [20]. Dual-color FISH was performed as in Ji et al. (1997) [34] with some modifications. Purified BAC DNA was labelled with biotin-16-dUTP or digoxigenin-11-dUTP by the BioNick Labelling System (Roche Diagnostics, Mannheim, Germany). Following post-hybridization washes, signals from the digoxigenin-labelled probes were detected by anti-digoxigenin-rhodamine, and directly from biotin-labelled probes by avidin-fluorescein. DAPI (4', 6-diamidino-2-phenylindole) (Sigma, St. Louis, MO) in an antifade solution, Vectashield (Vector, Burlingame, CA) was used to counterstain the chromosomes. Slides were examined under an Olympus BX51 fluorescence microscope. Chromosome and FISH signal images were captured using an Evolution VF CCD camera (Media Cybernetics, Silver Spring, MD) and merged using Image-Pro Express software.

BAC-end sequencing was performed by National Center for Gene Research, CAS, China. And the online software Primer3 was used to design the BAC-specific primers. Loci were genetic mapped on the genetic map by combination the recombination data among the BC_1 _population with the new map data [35].

## Authors' contributions

KW performed all the experiments and drafted the manuscript. WZG and TZZ conceived the study, participated in its design and coordination, drafted and revised the manuscript. All authors read and approved the final manuscript.
